# Road Performance Investigation on Fiber-Reinforced Recycled Cement Base Material

**DOI:** 10.3390/polym14194102

**Published:** 2022-09-30

**Authors:** Yongcheng Ji, Wenhao Ji, Ziyi Zhang, Rui Wang

**Affiliations:** School of Civil Engineering, Northeast Forestry University, Harbin 150040, China

**Keywords:** fiber cementitious materials, mechanical property, pavement structure, waste red brick, fiber microstructure

## Abstract

The characteristics of the materials used in early buildings in China have led to a large proportion of discarded red bricks among the construction waste generated by demolishing abandoned buildings. The application of red brick aggregate with a particle size ≤5 mm and red brick powder with particle size 0.125~0.75 mm (referred to as recycled brick powder) was studied in this study after the crushing of waste red brick in road structures. The research results will provide a theoretical basis for the whole-grain recycling of waste red brick aggregate. The aggregate of red brick with a particle size smaller than 2 mm was mixed with different amounts of cement soil and fiber to prepare a cement-stable binder for the sub-base material. The recycled brick powder of 0.125~0.75 mm was used to replace the quartz sand with different substitution rates. As pavement materials, different amounts of fiber were used to prepare fiber-reinforced recycled-brick-powder cementitious composites. The optimal mixing ratio of the two materials was evaluated from the mechanical properties. The results showed that the optimal mixing ratio of the cement-stable binder was as follows: waste-red-brick-aggregate content was 50%, cement content was 4%, and fiber content was 0.2%. The optimum ratio of fiber-reinforced recycled-brick-powder cementitious composites was determined to be as follows: the replacement rate of recycled brick powder is 25%, and the content of PVA fiber is 1%. The regression analysis was used to fit the equations between the fiber content and the 7d unconfined compressive strength and the tensile strength of the cement-stabilized binder for different red-brick-aggregate admixtures at 4% cement content. A scanning electron microscope was used to observe the failure modes of the fiber. The influence of failure modes, such as pulling out, fracture, and plastic deformation, on the mechanical properties was expounded.

## 1. Introduction

**Figure 1:**
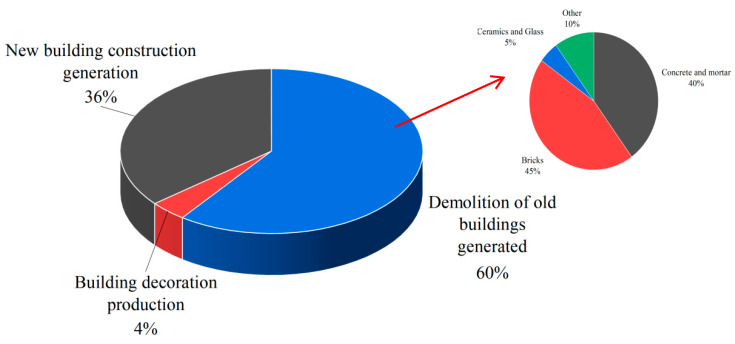
Proportion of various construction wastes.

The annual output of construction waste reached 3 billion tons and continued to show an increasing trend in 2020 [1]. In the past, influenced by economic development and traditional construction habits, many sintered clay red bricks were used in old buildings. However, in recent years, with the rapid development of urban–rural integration, a large amount of construction waste, mainly composed of red bricks, will be generated during the demolition and renovation of old rural houses, accounting for about 35–45% of the total construction waste [2]. The specific proportion is shown in Figure 1, below. At the same time, with the progress of road infrastructure projects, many fillers are needed, and plain-soil backfilling destroys cultivated land and occupies many land resources [3]. Therefore, the waste red brick is applied to the road backfilling material. It not only can effectively alleviate a large amount of construction waste produced yearly but also reduce a large amount of earth and rock required by the road infrastructure project.

Scholars at home and abroad have conducted extensive studies on recycling and using recycled red brick. Ren et al. [4] used orthogonal experiments to explore the preparation of recycled aggregate permeable concrete with recycled red brick aggregate of different particle sizes (5–10 mm and 10–20 mm) and different substitution rates (5%, 10%, 15%, and 20%). The results show that the mechanical properties and water permeability of recycled aggregate pervious concrete are better when the red brick replacement rate is 15% and the sand rate is 6%. The strength of recycled aggregate pervious concrete with 10–20 mm is lower than that with 5–10 mm, but the water permeability is better than that with 5–10 mm. Li [5] prepared cement mortar by replacing recycled concrete fine aggregates with recycled red bricks of a particle size less than 5 mm in different proportions. The results showed that each performance index of recycled fine aggregate decreased with the increase of red brick content. The water requirement of recycled cement mortar was linearly and positively correlated with the red brick content. The compressive and flexural strengths were reduced to different degrees, and the compressive strength of recycled cement mortar was highly linearly and negatively correlated with the red brick content. Sun [6] used red waste bricks as coarse aggregate for the preparation of concrete, which was analyzed in a compressive-strength experiment and abrasion-resistance experiment, and determined that the recycled concrete prepared from the coarse aggregate of recycled red bricks could meet the requirements. Kim [7] et al. used the broken red brick as coarse aggregate and prepared concrete with 0%, 30%, and 60% content, respectively. The results showed that the concrete with 30% coarse red brick aggregate performed similarly to ordinary concrete. Ji [8] et al. used discarded red bricks instead of natural aggregates. They combined them with steel fiber to establish a finite element model of steel-fiber/recycled-brick-aggregate concrete under uniaxial compression.

Scholars have also paid close attention to improved road structural materials. Zhou [9] conducted an indoor experiment by mixing construction waste of three granular combinations into expanded soil at 0%, 20%, 30%, 40%, and 50%. The results showed that, when the compaction degree meets the specification requirements, choosing a suitable combination of aggregate and mixing ratio and construction waste improved expansive soil satisfies the requirement of highway roadbed material strength and expansion rate of total demand. Xu [10] studied the characteristics of the unconfined compressive strength of soil–cement mixed with glass fiber under the freeze–thaw cycle. The study showed that the unconfined compressive strength of soil–cement could be improved when the mass fraction of glass fiber was 0.5%, and the strength was the highest when the length of glass fiber was 3 mm. Khiem [11] studied the influence of corn fiber content on the mechanical properties of soil–cement, found that the incorporation of corn fiber enhanced the compressive and tensile strength of soil–cement, and determined that the optimal incorporation range of corn fiber in soil–cement was 0.25~0.5%. Yu [12] et al. blended waste concrete:waste red bricks = 100%:0%, 50%:50%, and 10%:90% into cement-stabilized aggregates and conducted experimental studies at cement doses of 3%, 5%, and 7%, respectively. The results showed that using recycled aggregate as a road base was feasible. Li [13] et al., using construction waste as aggregate, verified the feasibility of applying recycled aggregate made of construction waste in subgrade through experiments such as unconfined compressive strength and California bearing ratio.

Cement concrete pavement is widely used because of its high strength, good integrity, and strong carrying capacity. However, it also has certain defects. For example, a certain degree of cracking and plate breaking will appear during use. In order to overcome the defects of the cement-concrete pavement, the researchers have made various improvements to the cement-based pavement materials. Li [14] studied the natural brucite mineral fiber and industrial-solid-waste fly ash in pavement concrete. The results show that the early performance of the pavement concrete is not apparent, but 28d-age-and-above strength, impact resistance, and toughness are significantly higher than those of the base group. Simultaneously, the incorporation of brucite fiber can significantly improve the durability of concrete. Ma [15] studied the influence rule of steel slag powder content on the road performance of a cement composite. The results showed that the incorporation of steel slag powder reduces the water consumption of standard consistency and prolongs the setting time of cement. When the mixing capacity of steel slag powder is less than 40%, the stability of the steel slag cement composite is qualified. The 28d compressive and flexural strengths showed a rising and decreasing trend with the increase of steel-slag-powder dosing, with a peak at 20% of steel-slag-powder dosing. Qi [16] used waterborne epoxy resin as a modifying agent and added an appropriate amount of fly ash to prepare the modified mortar for pavement materials. This researcher studied composite materials’ optimal mix ratio and road performance through a compressive test, anti-folding test, bond strength test, and anti-skid performance test. The results showed that fly ash mixed at 10% and waterborne epoxy resin mixed at 10% have the best mechanical properties, bonding strength, and anti-skid performance to meet the standard requirements. Qian [17] et al. showed that fiber-cement-based composite paving could reduce the thickness of pavement to a great extent compared with ordinary concrete pavement. It also effectively curbs the upward reflection crack expansion of the subgrade, and its fatigue life is more than double that of ordinary concrete pavement.

Red waste bricks are mainly applied as recycled aggregates for preparing cementitious materials and filling roadbeds. The red brick aggregates’ particle size is concentrated above 5 mm. However, waste red bricks will inevitably produce particles or dust with particle size ≤5 mm during the crushing process, which will cause secondary damage to the environment if not utilized. Therefore, in order to make a recycled red brick aggregate of all sizes that can be used, this study adopted the regeneration of the particle size of 5 mm or less red brick particles with different dosages mixed with soil, with 9 mm long polyvinyl alcohol (PVA) fiber [18]. A prepared cement-stabilizing binder was applied to the road sub-base, with an unconfined-compressive-strength test and split test as the evaluation index, to explore the influence of fiber, cement, and recycled red brick content on the mechanical properties of the stable inorganic binder. The recycled brick powder with a particle size of 0.075~0.125 mm was used to replace quartz sand with different substitution rates. Combined with 6 mm–long PVA fiber, the fiber-reinforced recycled-brick-powder cementitious composite was prepared with low cost, low carbon, and environmental protection. A mechanical-property test determined the optimal mixing amount of recycled brick powder and PVA fiber. The experiment results will provide a theoretical basis for fully recycling red waste bricks at all levels in the future and help achieve a “carbon peak” in 2030.

## 2. Experimental Program

### 2.1. Material Properties

**Figure 2:**
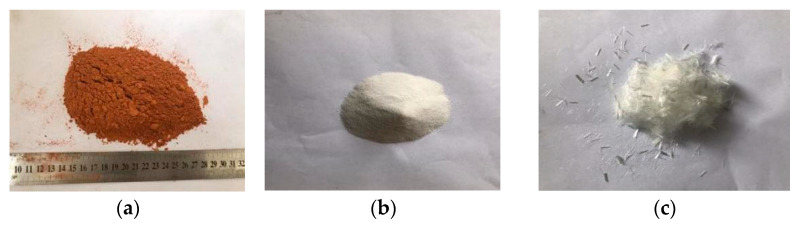

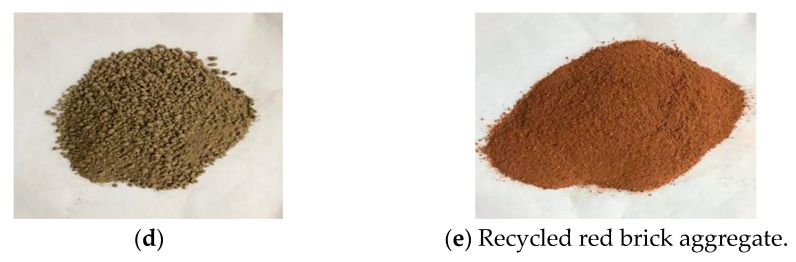
Test raw materials. (**a**) Recycled brick powder. (**b**) Quartz sand. (**c**) PVA fiber. (**d**) Plain soil. (**e**) Recycled red brick aggregate.

The raw materials used in the experiment include cement, fly ash, quartz sand, PVA fiber, waste red brick aggregate, plain soil, a water-reducing agent, and a thickening agent. The cement used in this experiment is P.O. 42.5 ordinary silicate cement, and its performance index is in line with GB175-2007 “Common Portland Cement” specification standard research, as shown in Table 1. The fly ash is the grade I fly ash produced by Shanxi Longhui Building Materials Co., LTD. The specific parameters of the material are shown in Table 2. The particle size of quartz sand is 120–200 mesh produced by Zhengzhou Hanhai Environmental Protection Technology Co., LTD. PVA fiber is produced by Shanghai Chenqi Chemical Technology Co., LTD. The specific parameters are shown in Table 3. The waste red brick aggregate is the waste red brick obtained from the demolition of a specific building in Harbin; it is obtained by crushing and screening. The plain soil is clay soil distributed from 1.5–3 m underground in Harbin city, and the specific indexes are shown in Table 4. The water-reduction rate of the polycarboxylic acid superplasticizer was 28.5%; hydroxypropyl methylcellulose thickening agent was used, at viscosity class 200000. The water used in the experiment is Harbin ordinary tap water. The red brick aggregate with particle size ≤5 mm, recycled brick powder with a particle size of 0.075~0.125 mm, plain soil, quartz sand, and PVA fiber are shown in Figure 2.

### 2.2. Proportioning Design and Test Method

#### 2.2.1. Compaction Test

##### Proportioning Design

The compaction test explored the samples’ optimal moisture content and maximum dry density under different mixing ratios. With the content of recycled red brick aggregate (0%, 50%, and 100%) and cement content (3%, 4%, and 5%) as the research variables, five groups of samples with a moisture content of 10%, 12%, 14%, 16%, and 18% were prepared for each group, so 45 compaction tests were needed. Table 5 shows the specific mix ratio of the compaction test.

##### Experimental Method

The compaction test can analyze the compaction characteristics of the soil, and the variation of the moisture content of the soil with the dry density under certain fixed conditions can be obtained to determine the optimum moisture content and the maximum dry density value of the plain soil used. This test adopts a heavy compaction test, the instrument used for the laboratory multifunctional electric compaction instrument. The height of the compaction cylinder is 12.7 cm, the volume is 997 cm^3^, the hammer is 4.5 kg, the falling height is 45 cm, and the compaction times of each layer are 27 times.

**Figure 3:**
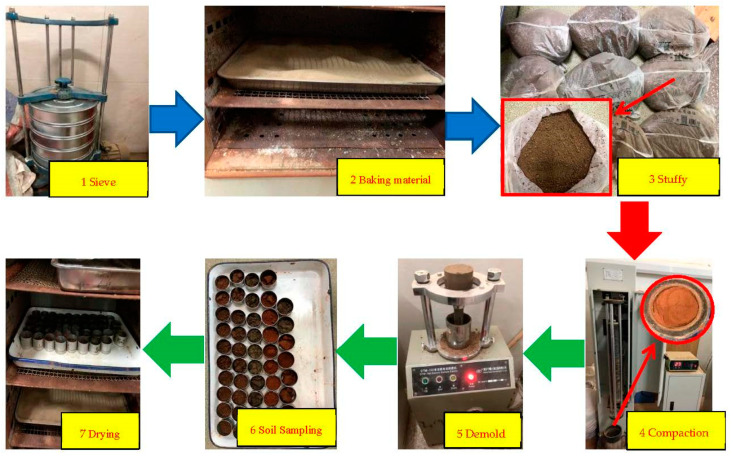
Compaction test process.

Soil samples and waste brick aggregates were sieved. Soil samples and red brick aggregate below 5 mm were taken, dried, and added with different moisture (in 2% water content increments); they were then mixed well and set for 12 h. Apply a layer of petroleum jelly on the compaction cylinder, and tightly fix the compaction cylinder and the compaction instrument. Pour the preprepared compacted material into the compaction cylinder in five times. After 27 times of compaction, the surface of the soil sample should be “brushed” to make the five layers of the soil sample closely connected. After the compaction is completed, the soil height on the top surface of the compaction cylinder is measured. If the height is less than 5 mm, the compaction is qualified. The top and bottom surfaces of the compacted soil were cut flat to make the volume of the soil the same as the volume of the compaction cylinder. The soil samples were removed from the compaction cylinder with a multifunctional electric demold machine, and two soil samples were taken from the center to measure the moisture content. The above steps were repeated for all ratios and water-content specimens, and the specific operation flowchart is shown in Figure 3.

#### 2.2.2. Tests for 7d Unconfined Compressive Strength and Splitting Strength

##### Proportioning Design

The 7d unconfined compressive strength and splitting strength are essential indicators to verify the performance of inorganic bonded stabilized materials as sub-base materials. Therefore, based on the best moisture content and maximum dry density of each ratio obtained from the compaction test, each ratio was mixed with PVA fiber (0%, 0.1%, 0.2%, and 0.3%) to prepare the PVA-fiber bond-test specimens. The amount of each material was based on Formulas (1)–(5), and the specific ratio is shown in Table 6.

The standard mass of each specimen is calculated as follows:

(1) $$m_{0}=v\times \rho_{\max}\times (1+\omega_{\mathrm{opt}})\times \gamma$$

The total mass of dry material (dry soil + cement) for each specimen is calculated as follows:

(2) $$m_{1}=\frac{m_{0}}{1+\omega_{\mathrm{opt}}}$$

The mass of inorganic binding material (cement) in each specimen is calculated as follows:

(3) $$m_{2}=m_{1}\times \alpha$$

The dry soil mass in each specimen is calculated as follows:

(4) $$m_{3}=m_{1}-m_{2}$$

The water added to each specimen is calculated as follows:

(5) $$m_{\omega }=(m_{2}+m_{3})\times \omega_{\mathrm{opt}}$$

where v is the volume of the specimen (cm^3^), taken as 98.125; $\rho_{\max}{\;\mathrm{and}\;\omega }_{\mathrm{opt}}$ are the optimum moisture content (%) and the maximum dry density (g/cm^3^), respectively; γ  is used for the mix compaction standard (%). According to the “Technical Guidelines for Construction of Highway Roadbases” (JTG/T F20-2015), the requirements for the construction of highway sub-base compaction should not be less than 95%, and 95% was used in this study. Moreover, α is the content of inorganic binder (%).

##### Experimental Method

**Figure 4:**
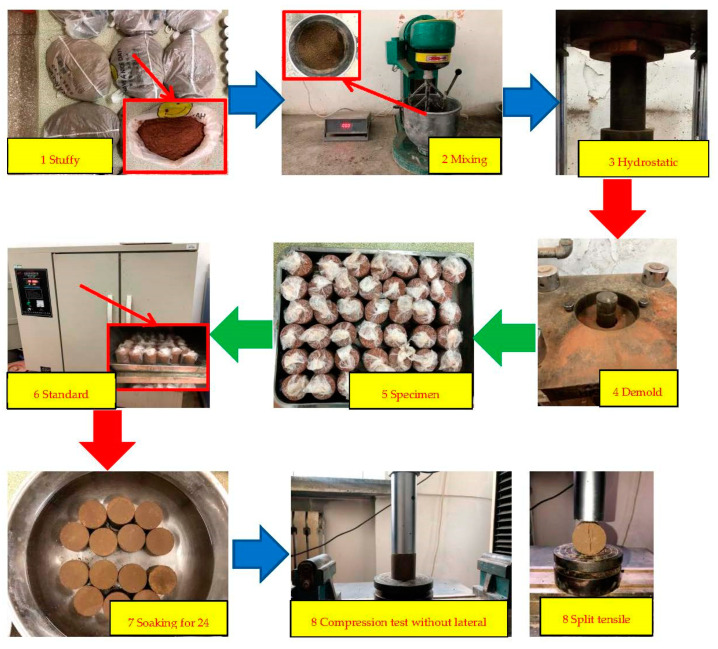
Flowchart for 7d unconfined compressive test and splitting tensile test.

The specimen preparation of the 7d-unconfined-compressive-strength test and splitting-strength test was carried out by taking waste red brick aggregate and plain soil particles below 2 mm. The test followed the “Test Method of Materials Stabilized with Inorganic Binders for Highway Engineering” (JTG E51-2009), from the preparation of specimens to the health preservation. The sample size was a 50 × 50 mm cylinder, and the prepared raw materials were mixed evenly, and then the material was stuffed for 12 h. After the stuffy material was finished, the corresponding cement and PVA-fiber quality were added. Next, a planetary cement mortar mixer fully and evenly mixed the material. The specimen was hydrostatically formed by the pressure/demolding tester, with a range of 300 KN. After demolding, the specimens were wrapped with plastic and placed in a standard constant temperature and humidity curing box for seven days before the test. The day before the end of the regimen, the specimens should be immersed in water for 24 h. Next, a universal testing machine performed the unconfined-compressive-strength and unsplit-tensile-strength tests. The loading rate of the press was controlled to be 1 mm/min, and the maximum pressure at the failure of the specimen was recorded. The seven-day unconfined compressive strength and splitting tensile strength of the specimen were calculated according to Equations (6) and (7). A total of eight steps are required, and the specific test operation process is shown in Figure 4.

The 7d unconfined compressive strength is calculated according to the following equation:

(6) $$R_{c}=\frac{P}{A}=\frac{4P}{{\pi D}^{2}}$$

where $R_{c}$ is the compressive strength of the sample under different mix ratios, MPa; P is the maximum pressure at the time of specimen failure, N; A is the cross-sectional area of the specimen, mm^2^; and D is the diameter of the specimen, mm.

The splitting tensile strength is calculated according to the following equation:

(7) $$R_{i}=\frac{2P}{\pi \mathrm{dh}}=0.01273\frac{P}{h}$$

where $R_{i}$ is the splitting tensile strength of the sample at different mix ratios, MPa; P is the maximum pressure at the time of specimen failure, N; d is the diameter of the specimen, mm; and h is the height of the specimen soaked in water, mm.

#### 2.2.3. Mechanical Properties of Fiber-Reinforced Cementitious Materials

##### Proportioning Design

This experiment studied the effect of the substitution rate of recycled brick powder replacing quartz sand and the volume of PVA-fiber content on the fiber-reinforced recycled-brick-powder cementitious composites’ performance. Based on the water–binder ratio of 0.32 and the rubber–sand ratio of 0.36, the substitution rates of recycled brick powder replacing quartz sand are 0%, 25%, 50%, 75%, and 100%, respectively. The fiber volume content was 0%, 1.0%, 1.5%, and 2.0%, respectively. A total of 20 groups of mix ratios were designed by using the control variable method, and the specific mix ratio is shown in Table 7.

##### Experimental Method

**Figure 5:**
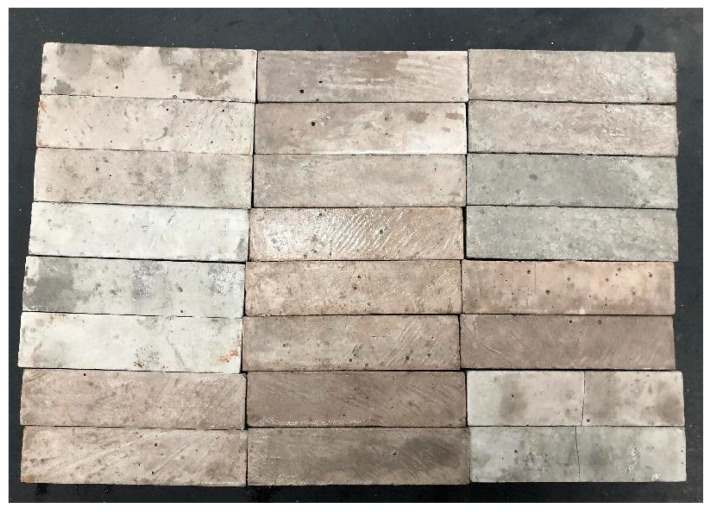
Specimens of fiber-reinforced recycled-brick-powder cementitious composite.

Cement, fly ash, quartz sand, and recycled brick powder were added to the mixing tank. Stir for 120 s so that it is fully mixed. Then add some water mixed with the admixture and stir for 120 s, and add the remaining water and stir for 120 s. After stirring evenly, add PVA fiber, dispersing manually and stirring for 180 s until no fiber agglomerates. Next, the mold is filled in layers, formed by vibration, covered with plastic film, cured for 24 h, and then demolded, as shown in Figure 5. Follow up with mechanical test conditions after 28 days of standard curing.

**Figure 6:**
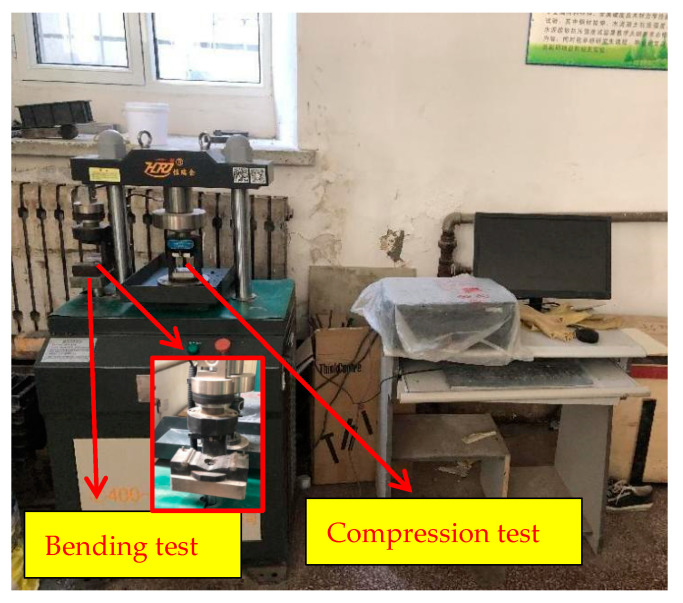
Compression- and bending-test machine.

**Figure 7:**
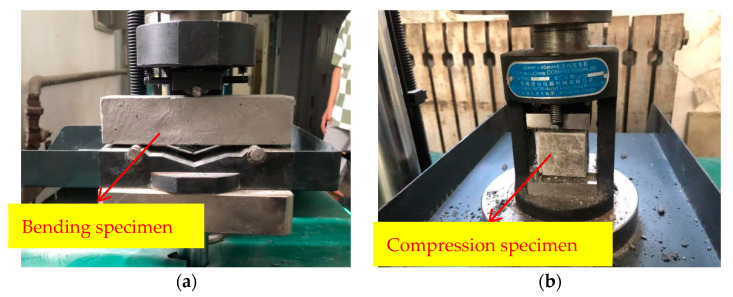
Mechanical test. (**a**) Bending test. (**b**) Compression test.

The compressive and flexural tests were conducted by using the YAW-300H testing machine produced by the Jinan Hengruijin Company, as shown in Figure 6. The test procedure was carried out according to the “Test Method of Cement Mortar Strength (ISO method)” (GB/T 17671-2020), and the loading speed of the flexural test was 50 N/s ± 10 N/s. The two half sections after breaking were taken out for a compressive test, and the loading rate was 2400 N/s ± 200 N/s. Therefore, the specimen size for the flexural test was 40 mm × 40 mm × 160 mm, and the size was 40 mm × 40 mm × 40 mm for the compressive test, as shown in Figure 7.

### 2.3. Microscopic Experiments

**Figure 8:**
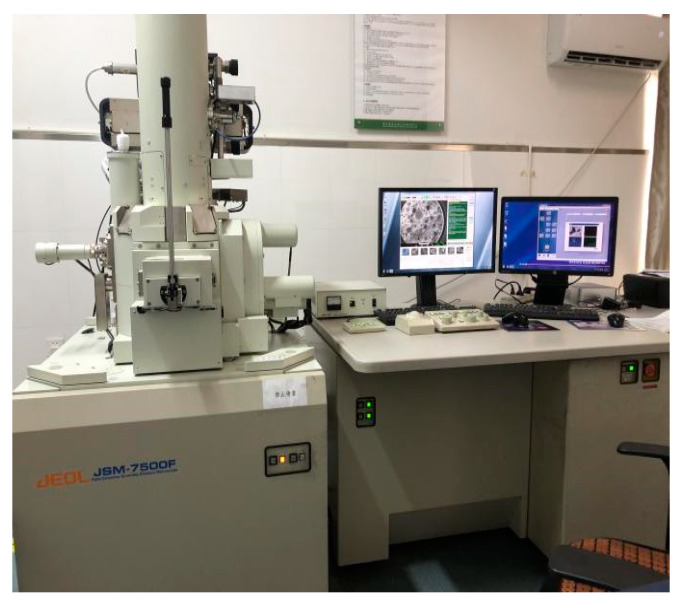
Scanning electron microscope.

The SEM test was carried out in the Analysis and Testing Center Laboratory of Northeast Forestry University, using JMF-7500F (Cold Field Emission Scanning Electron Microscope), as shown in Figure 8. The surface of the flat sheet sample was first gilded to improve its electrical conductivity. After the gold injection, the charge plate was fixed under the electron microscope for scanning.

## 3. Experimental Results and Analysis

### 3.1. Compaction Test

The compaction test is performed to obtain the maximum dry density and optimal moisture content of the test sample, provide the basis for the design of the raw-material mix ratio of the pavement structure layer, and guide and control the construction. The samples with different proportions were subjected to a compaction test according to the method in “Test Method of Materials Stabilized with Inorganic Binders for Highway Engineering” (JTG E51-2009). The maximum dry density and optimal moisture content of each mixture ratio are shown in Table 8.

**Figure 9:**
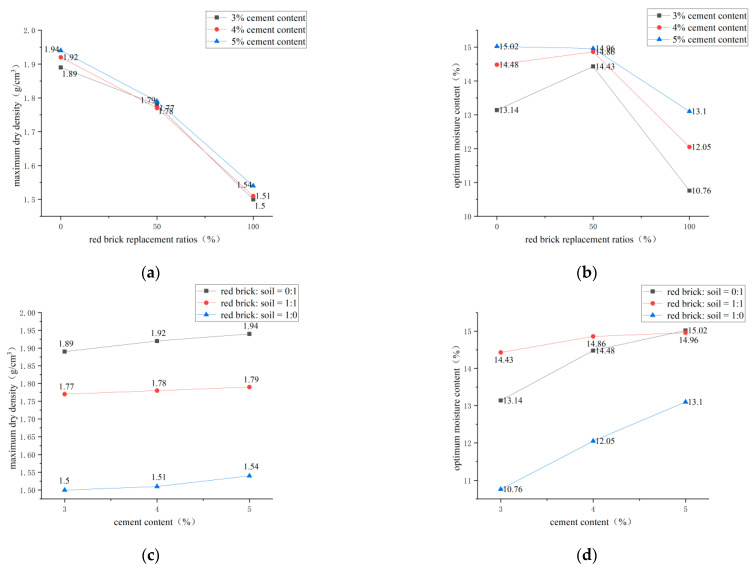
Compaction test analysis. (**a**) Maximum dry density and red-brick-aggregate replacement ratio. (**b**) Optimal moisture content and red-brick-aggregate replacement ratio. (**c**) Cement content and maximum dry density under different ratios. (**d**) Cement content and optimal moisture content under different ratios.

It can be seen from Figure 9a that, when the cement content is constant, the maximum dry density of the specimen decreases with the increase of the red-brick-aggregate content. This is because the appearance of the red brick particles is sharp, and the gap between the particles is more significant than the plain soil. In addition, the hardness is higher than the plain soil, so the density of the red brick aggregate is less than that under the same compaction action of the plain soil. As seen in Figure 9b, the optimal moisture content of the sample increases first. It then decreases with the incorporation of red brick aggregate, reaching the maximum value when the incorporation of red brick aggregate is 50%. When the red-brick-aggregate content is at 50%, soil particles can be filled with red brick particles, which will lock more water, but when the red-brick-aggregate content is greater than 50%, the red brick aggregate dominates in the sample. Due to the nature of the red brick aggregate, there is less water absorption, and the ability to lock water is weak, so the optimal moisture content will gradually decline after the red brick aggregate exceeds 50%. As can be seen from Figure 9c,d, when the red brick aggregate’s mix ratio is certain, the maximum dry density and the optimal moisture content both increase with the increasing cement content. The increase of the optimum water content may be due to the chemical reaction between the cement and plain soil particles, which consume water in the sample during the reaction process, leading to the increase of the optimum water content. The increase in the maximum dry density occurs because the cement is mixed into the sample at different doses, equivalent to replacing some plain soil or red brick particles with cement. The relative density of cement is higher than that of plain soil and red brick, so the incorporation of cement increases the maximum dry density of the sample [19].

### 3.2. Tests for 7d Unconfined Compressive Strength and Splitting Strength

Cement-stabilized soil is generally used as the sub-base of road construction. In the design code of asphalt pavement or concrete pavement structure, the strength of the road’s sub-base has specific strength requirements. For example, the size of the 7d unconfined compressive strength can reflect the limited strength of the cement-stabilized soil resistance to the axial pressure. In contrast, the splitting strength will reflect the indirect tensile properties of the pavement base. The compressive strength and split tensile strength of each mixing ratio test are listed in Table 9.

#### 3.2.1. Effect of Cement Content on 7d Unconfined Compression Strength and Splitting Tensile Strength

**Figure 10:**
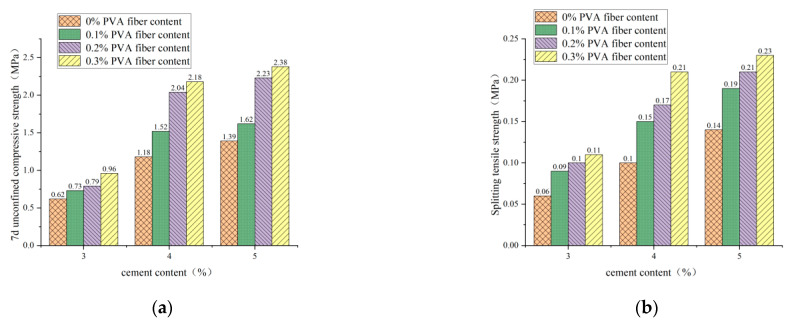
Cement admixture with 7d unconfined compressive strength and splitting tensile strength for plain soil:red brick aggregate = 1:0. (**a**) Cement content and 7-day unconfined compressive strength. (**b**) Cement content and splitting tensile strength.

**Figure 11:**
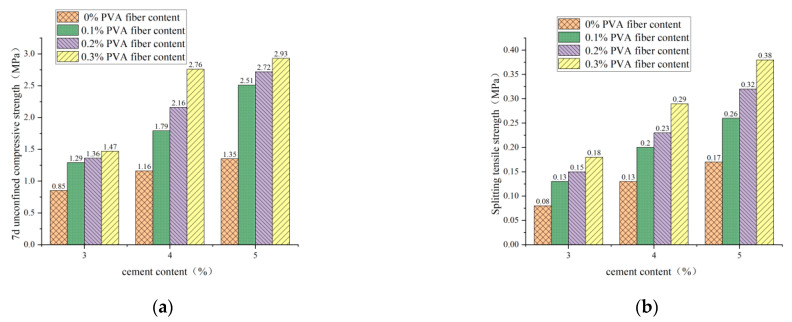
Cement admixture with 7d unconfined compressive strength and splitting tensile strength for plain soil:red brick aggregate = 1:1. (**a**) Cement content and 7-day unconfined compressive strength. (**b**) Cement content and splitting tensile strength.

**Figure 12:**
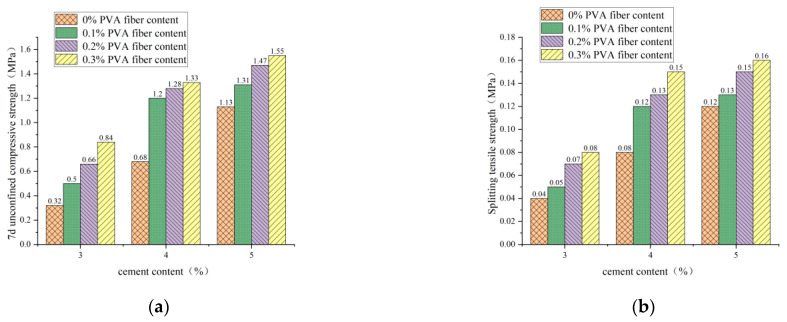
Cement admixture with 7d unconfined compressive strength and splitting tensile strength for plain soil:red brick aggregate = 0:1. (**a**) Cement content and 7-day unconfined compressive strength. (**b**) Cement content and splitting tensile strength.

Figure 10, Figure 11 and Figure 12 show the influence of cement content on 7d unconfined compressive strength and splitting tensile strength under three conditions: plain soil:red brick aggregate = 1:0, 1:1, and 0:1, respectively. It can be seen from the figure that, under a certain amount of fiber and red brick aggregate, the unconfined compressive strength and splitting tensile strength of 7d show an upward trend with the increase of cement content. However, cement content from 3% to 4% and from 4% to 5% caused by the strong growth trend is different. Cement content increased from 3% to 4%, 7d unconfined compression strength and splitting tensile strength increased by more than 60%, and the mix ratio increased by more than 100%. For example, for a plain soil:red brick aggregate ratio of 0:1, with a PVA-fiber incorporation of 0%, the compressive strength and tensile strength increased by 120% and 100%, respectively. When the cement content increased from 4% to 5%, the 7d unconfined compression strength and splitting tensile strength increased by less than 30%. For example, when the plain soil:red brick aggregate = 1:0, after a PVA-fiber incorporation of 0.3%, its compressive strength and tensile strength only increased by 9.2% and 9.5%, respectively. This is because the reaction of cement and water produces many cement hydration products and soil particles and brick aggregate to form a three-dimensional mesh of cement slurry gradually. The condensation between the reactants can make the particles close and make the specimen more compact, thus improving the 7d unconfined compression strength and splitting tensile strength of the specimen [20]. However, the strength improvement is not apparent as the cement content increases from 4% to 5%. For the consideration of economy, the cement content is 4%, and the strength meets the requirements of the code.

#### 3.2.2. Influence of PVA-Fiber Content on 7d Unconfined Compressive Strength and Splitting Tensile Strength

**Figure 13:**
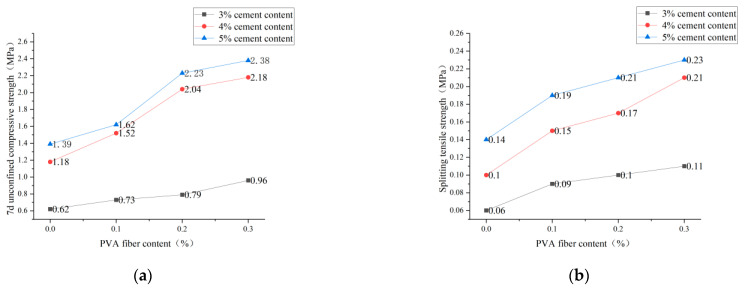
PVA-fiber content with 7d unconfined compressive strength and splitting tensile strength for plain soil:red brick aggregate = 1:0. (**a**) PVA-fiber content and 7d unconfined compressive strength. (**b**) PVA-fiber content and splitting tensile strength.

**Figure 14:**
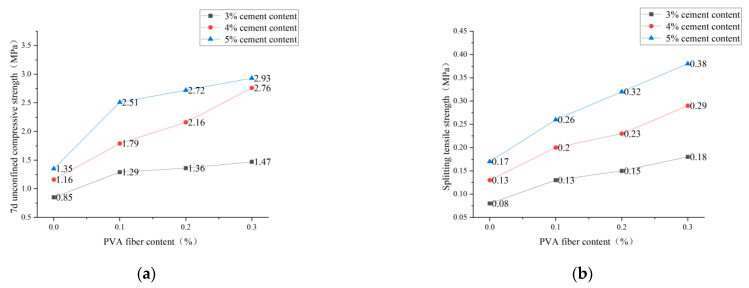
PVA-fiber content with 7d unconfined compressive strength and splitting tensile strength for plain soil:red brick aggregate = 1:1. (**a**) PVA-fiber content and 7d unconfined compressive strength. (**b**) PVA-fiber content and splitting tensile strength.

**Figure 15:**
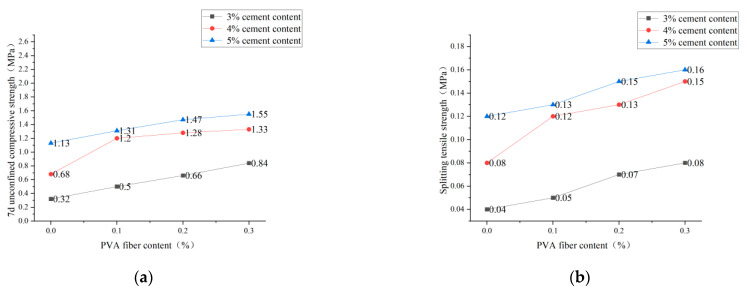
PVA-fiber content with 7d unconfined compressive strength and splitting tensile strength for plain soil:red brick aggregate = 0:1. (**a**) PVA-fiber content and 7d unconfined compressive strength. (**b**) PVA-fiber content and splitting tensile strength.

It is clear from Figure 13, Figure 14 and Figure 15 that the incorporation of PVA fibers positively affects the 7d unconfined compressive strength and splitting strength of the specimens at any red-brick-aggregate admixture and cement content. When the amount of PVA-fiber incorporation is not greater than 0.3%, the 7d of unlimited compressive strength and split tensile strength gradually increase with the increase of PVA-fiber incorporation. PVA fiber improves the 7d unconfined compressive strength because the fibers are distributed in a three-dimensional disorderly direction in the cement-stabilized soil, so there will be fibers with the transverse distribution. Considering the deformation coordination relationship, when subjected to axial pressure, the specimen will be subjected to transverse tensile force, and the fibers with transverse distribution will also be subjected to transverse tensile force. Since fibers will play the constraint role of bearing tensile force, the fiber-and-cement-stabilized soil will jointly bear the horizontal tension. Thus, the ultimate axial pressure that the test block can bear will also increase accordingly, thus improving the unconfined compressive strength of the specimen [21]. Fiber-enhanced splitting tensile strength: ① PVA fiber itself has an elastic modulus and tensile strength that are higher than those of cement-stabilized soil. The fiber can share a large part of the load when the cement stabilizes the soil load. After the cracking load, the fiber between the cracks plays a bridge role, making the specimen continue to load [22]. ② The surface extrusion plastic deformation of PVA fiber was influenced by soil particles and red brick aggregate from the micro perspective. When the test is under external load and deformation, the surface roughness of PVA fiber increases to overcome greater friction, thus improving the splitting tensile strength.

**Figure 16:**
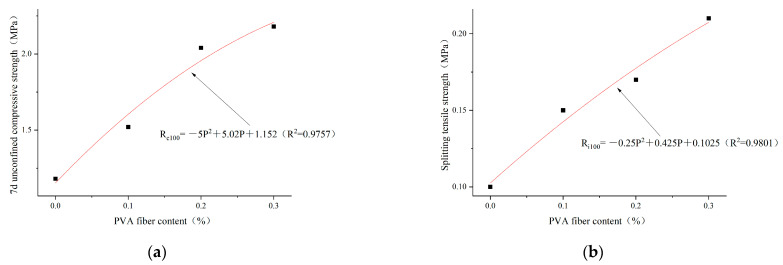
Regression curves of PVA content and 7d unconfined compressive strength and splitting tensile strength for plain soil:red brick aggregate = 1:0, cement content 4%, and PVA-fiber content within 0.3%. (**a**) Regression curve of PVA-fiber content and 7d unconfined compressive strength. (**b**) Regression curve of PVA-fiber content and splitting tensile strength.

**Figure 17:**
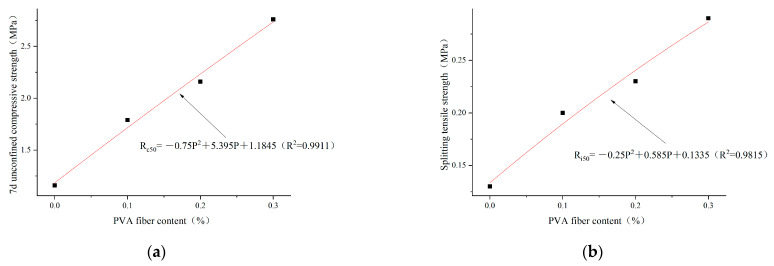
Regression curves of PVA content and 7d unconfined compressive strength and splitting tensile strength for plain soil:red brick aggregate =1:1, cement content 4%, and PVA-fiber content within 0.3%. (**a**) Regression curve of PVA-fiber content and 7d unconfined compressive strength. (**b**) Regression curve of PVA-fiber content and splitting tensile strength.

**Figure 18:**
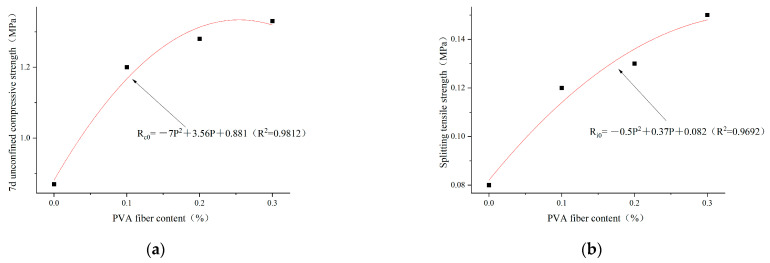
Regression curves of PVA content and 7d unconfined compressive strength and splitting tensile strength for plain soil:red brick aggregate = 0:1, cement content 4%, and PVA-fiber content within 0.3%. (**a**) Regression curve of PVA-fiber content and 7d unconfined compressive strength. (**b**) Regression curve of PVA-fiber content and splitting tensile strength.

Based on the analysis results in Section 3.2.1, the optimal cement content selected is 4%. The regression analysis is carried out according to the test results of unconfined compressive strength and splitting tensile strength in 7 days, as shown in Figure 16, Figure 17 and Figure 18. The relationships between PVA-fiber content and 7d unconfined compressive strength and splitting tensile strength were obtained, respectively, under the conditions of cement content of 4%; plain soil:red brick aggregate = 1:0, 1:1, and 0:1; and PVA-fiber content being within 0.3%, as shown in Equations (8a,b)–(10a,b).

The cement content is 4%. The ratio of plain soil to red brick aggregate is 1:0. The content of PVA fiber is 0~0.3%. The relationship among PVA-fiber content, 7d unconfined compressive strength, and splitting tensile strength can be expressed as follows:

(8a) $$R_{c100}=-5P^{2}+5.02P+1.152\;(R^{2}=0.9757)$$

(8b) $$R_{i100}=-0.25P^{2}+0.425P+0.1025\;(R^{2}=0.9801)$$

The cement content is 4%. The ratio of plain soil to red brick aggregate is 1:1. The content of PVA fiber is 0~0.3%. The relationship among PVA-fiber content, 7d unconfined compressive strength, and splitting tensile strength can be expressed as follows:

(9a) $$R_{c50}=-0.75P^{2}+5.395P+1.1845\;(R^{2}=0.9911)$$

(9b) $$R_{i50}=-0.25P^{2}+0.585P+0.1335\;(R^{2}=0.9815)$$

The cement content is 4%. The ratio of plain soil to red brick aggregate is 0:1. The content of PVA fiber is 0~0.3%. The relationship among PVA-fiber content, 7d unconfined compressive strength, and splitting tensile strength can be expressed as follows:

(10a) $$R_{c0}=-7P^{2}+3.56P+0.881\;(R^{2}=0.9812)$$

(10b) $$R_{i0}=-0.5P^{2}+0.37P+0.082\;(R^{2}=0.9692)$$

where R_c_ and R_i_ represent the 7d compressive strength and splitting tensile strength, respectively, MPa; and P represents the content of PVA fiber, %.

According to Formulas (8)–(10), the ratios of plain soil to red brick aggregate are 1:0, 1:1, and 0:1 when the cement content is 4%. PVA-fiber content is 0~0.3% and has a quadratic function relationship with the 7d unconfined compressive strength and splitting tensile strength. Within the PVA-fiber-content test range, the ratios of plain soil to red brick aggregate are 1:0 and 1:1 when the cement content is 4%. The strength is positively related to the PVA-fiber content, and the optimal PVA-fiber content is 0.3%. However, the ratio of plain soil to red brick aggregate is 0:1, and the PVA-fiber content is 0~0.3%. The highest 7d unconfined compressive strength is obtained when the PVA-fiber content is 0.25%, and the highest splitting tensile strength occurs when the PVA-fiber content is 0.3%. Combined with 7d unlimited compressive strength and tensile strength of split, the ratios of plain soil to red brick aggregate are 1:0, 1:1, and 0:1. When the content of PVA fiber is within 0.3%, the optimal PVA-fiber content is 0.3%, 0.3%, and 0.25%, respectively. Therefore, a positive correlation is found between the intensity and PVA-fiber incorporation with PVA-fiber incorporation ≤0.25%, which is not affected by the amount of waste red brick aggregate.

#### 3.2.3. Influence of Red-Brick-Aggregate Content on 7d Unconfined Compressive Strength and Splitting Tensile Strength

**Figure 19:**
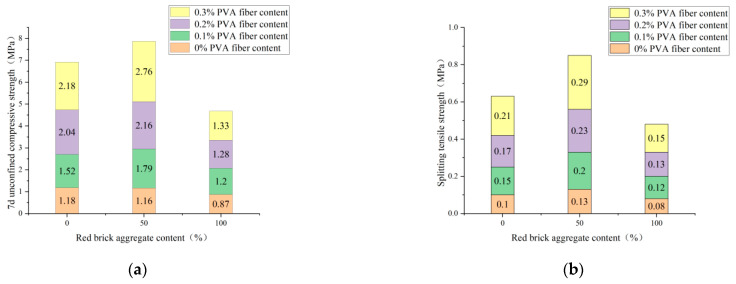
Red-brick-aggregate admixture with 7d unconfined compressive strength and splitting tensile strength at 4% cement admixture and different PVA-fiber content levels. (**a**) Red-brick-aggregate content and 7d unconfined compressive strength. (**b**) Red-brick-aggregate content and splitting tensile strength.

As seen from Figure 19, when the cement mixture is 4%, the 7d unconfined compressive strength and splitting tensile strength show a trend of first increasing and then decreasing with the increase of red-brick-aggregate incorporation, which is not affected by the fiber incorporation. When the amount of red brick aggregate is 50% and the fiber mixture is 0.3%, the 7d unconfined compressive strength and splitting tensile strength reach the maximum value, and the amount of red brick continues to increase, leading to a rapid decline in strength. The effect of fiber incorporation on strength was mentioned in the previous section. The more the incorporation is within 0.3%, the higher the intensity. When the red brick aggregate is 50% mixed, both the red brick particles and the plain soil particles have previously filled each other, increasing the compactness of the sample and, thus, increasing the strength. When the red brick aggregate increases, the red brick particles will dominate the specimen. Because the cohesion of the red brick particles is less than that of the plain soil particles, the strength drops sharply. Thus, the best amount of red brick aggregate is 50%.

In summary, combined with the “Technical Guidelines for Construction of Highway Roadbases” (JTG/T F20-2015) for highways and primary highways, light traffic road sub-grade 7d unconfined compressive strength requirements for 2~4 MPa. Within the scope of the experimental study, the strength of the test groups with red-brick-aggregate content ≤50%, cement content ≥4%, and fiber content ≥0.2% were all in line with the formal study and had a specific splitting tensile strength. Therefore, the optimal mixing ratio is 50% of the waste red brick aggregate, 4% cement, and 0.2% fiber considering the strength and material cost.

### 3.3. Mechanical Properties of Fiber-Reinforced Recycled Brick Powder Cementitious Composites

The most basic evaluation index of engineering materials is strength. The fiber-reinforced recycled-brick-powder cementitious composites must meet the strength design requirements to be applied to the road pavement. For cement concrete pavement, the pavement is bent when damaged, and flexural strength is a crucial factor to be considered when designing the pavement. Therefore, the compressive and flexural strength are two indispensable indexes to evaluate the road performance of fiber reinforced recycled brick powder cementitious composites. Therefore, a strength test was carried out on the specimens of each mixing ratio, and the test results are shown in Table 10.

**Figure 20:**
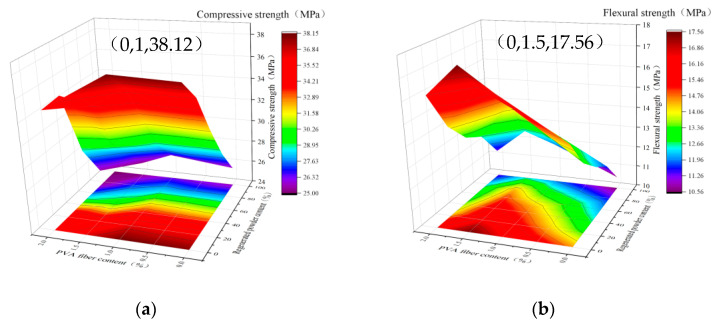
Three-dimensional surface with projection. (**a**) PVA-fiber volume admixture and recycled-brick-powder mass admixture with compressive strength. (**b**) PVA-fiber volume admixture and recycled-brick-powder mass admixture with flexural strength.

Figure 20 establishes the three-dimensional surface plots of the two variables of PVA content and recycled-brick-powder content with the mechanical properties of fiber-reinforced recycled-brick-powder cementitious composites. Figure 19a,b show the relationship between PVA fiber and recycled-brick-powder content with compressive and flexural strength, respectively. According to the color depth of the projection area, it can be seen that when the mass content of recycled brick powder is 0%, and the volume content of PVA is 1%, the fiber-reinforced recycled brick powder cementitious composites compressive strength reaches the highest, at 38.12 MPa, recorded as (0,1,38.12). The highest flexural strength of fiber-reinforced recycled-brick-powder cementitious composites reached 17.56 MPa when the dose of recycled brick powder was 0% and the volume dose of PVA was 1.5%, which was recorded as (0,1.5,17.56).

As seen from the color of the projection area in Figure 20a, the general trend of the whole surface graph shows a downward trend from the lower right corner to the upper left corner. It indicates that, with the increase of the mass content of recycled brick powder and the volume content of PVA fiber, the fiber-reinforced recycled-brick-powder cementitious composites’ compressive strength shows an overall downward trend. However, within a specific range in the lower right corner of the projection area, the color shades are relatively close, and the point (0,1) shows the deepest color of the entire projection area. It shows that, under a certain amount of PVA, recycled brick powder has little influence on the fiber-reinforced recycled-brick-powder cementitious composites compressive strength in a small range of mixing amounts. When the mixing amount of recycled brick powder is 25%, the compressive strength decreases by about 5–8%, and even part of the mixing ratio shows a strength increase. The recycled red brick aggregate has large porosity and low hardness. It often produces more cracks internally during the crushing process of preparing recycled brick powder. When the mass admixture of recycled brick powder is less than 25%, the recycled brick powder has a specific volcanic ash activity [23]. It can react with the hydration product CH to produce gelling products, which can play a good filling role and alleviate the material defects of the recycled brick powder itself. However, when the content of recycled brick powder exceeds 25%, the pozzolanic reaction of recycled brick powder is not enough to make up for the defects of the material itself. Thus, the strength will decrease. In the case of a certain amount of recycled brick powder, the fiber-reinforced recycled-brick-powder cementitious composites compressive strength showed a trend of increasing and then decreasing with the increase of PVA-fiber volume content and reached the maximum value when the volume dosing of PVA fiber was 1%. When the specimen is subjected to vertical load, the compression specimen is complete, and the bearing surface drops slightly. There is no breakage, and the specimen has a slight transverse displacement. A small amount of fiber will limit the transverse displacement of the specimen to share part of the axial pressure, thus improving the specimen’s compressive strength. However, when the fiber content exceeds the optimal amount, the fiber will appear to clump, resulting in uneven fiber distribution. The high fiber content will lead to the introduction of increased bubbles, and the increased air content in the specimen will harm the compactness, thus reducing the compressive strength [24].

As shown in Figure 20b, the projected color gradually becomes lighter from the lower part to the upper part of the projected area, indicating that the flexural strength shows a decreasing trend with the increase of the mass content of the recycled brick powder. From the analysis of the projection area’s left and right directions, the line’s color at the point where the fiber volume content is 1.5% is darker than that of the left and right sides. Therefore, the three-dimensional surface graph formed by the volume content of PVA fiber, the mass content of recycled brick powder, and the flexural strength presents a “ridge” shape. It indicates that the fiber-reinforced recycled-brick-powder cementitious composites flexural strength increases first and decreases with the increase of PVA—fiber volume content. It can be seen from the color change in the figure that the increase of flexural strength caused by PVA—fiber volume content from 0% to 1% is more evident than that caused by 1% to 1.5%. The micro-cracks of different sizes are generated inside the cementitious material, and a large stress concentration is generated near the micro-cracks during tension. It can be explained by the brittleness of the cementitious material itself and the self-shrinkage in the process of setting and hardening [25]. When the volume content of PVA fiber is less than 1.5%, the PVA—fiber incorporation reduces the stress intensity factor at the crack tip. It can fully play the connection role in the early stage of fracture emergence, ease the stress concentration intensity at the fracture tip, and limit the width of the fracture, thus improving the flexural strength [26]. When the volume content of PVA fiber exceeds 1.5%, the excessive fiber will increase the fiber interface in cement-based composites. However, an excessive interface between fiber and slurry will occupy a large amount of cement slurry, which will greatly slow down the mobility of the matrix and increase the material’s porosity, thus leading to the increase of initial defects inside the specimen. For example, cavitation caused by low vibration, initial cracks between fiber and interface, and the interface between fibers will adversely affect the matrix’s compactness, reducing the component’s flexural strength.

According to the “Specification for Design of Highway Cement Concrete Pavement” (JTG-D40-2011), the standard value of flexural tensile strength of cement concrete pavements for extremely heavy, extra heavy, and heavy traffic loads is ≥ 5.0 MPa, and the corresponding compressive strength is C35. Therefore, considering compressive strength, flexural strength, and economic and environmental protection, the best substitution rate of recycled brick powder for quartz sand in fiber-reinforced recycled-brick-powder cementitious composites is 25%, and the best mixing amount of PVA fiber is 1%.

### 3.4. Micro-Analysis

**Figure 21:**
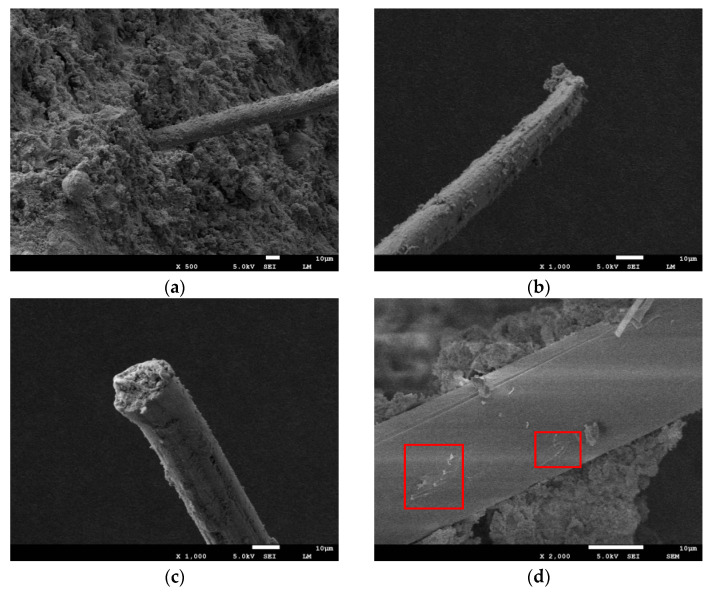
SEM image of PVA fiber. (**a**) SEM chart. (**b**) Pull-out. (**c**) Fracture. (**d**) Plastic damage.

Figure 21 shows PVA fibers’ overall appearance and morphology and three types of destructive patterns of samples under a scanning electron microscope. Firstly, it can be seen in Figure 20b–d that a large number of irregular particles are wrapped on the surface of the fiber, and this improves the roughness of the surface of the PVA fiber, increases the friction between the fiber and the matrix, and thus improves the strength of the specimen. Figure 20a shows the appearance of the PVA fiber pulled out from matrix morphology; part of the hydration products adhered to the fiber’s surface and had serious scraping marks. The closer to the end, the smaller the diameter of the fiber, and the more scraping needed. This suggests that fiber is pulled up after a long distance between sliding [27], and cumulative scraping is severe, leading to a decrease in the diameter. The friction force generated by the fiber during sliding helps to share part of the axial pressure, thus improving the strength of the specimen. Figure 20b is a microscopic photo of a PVA-fiber fracture. The section is uneven. Compared to Figure 20a, the side of the fiber was scraped to a lesser extent, indicating that the fiber did not go through a long distance of sliding. However, under the action of external forces, fiber is constantly stretched by the role of external tensile stress. When the external stress exceeds the ultimate tensile strength that the fiber can withstand, it will be directly pulled off. As the ultimate tensile strength of PVA fibers is much greater than that of cementitious materials, it will require more axial pressure to pull off the fibers, so a certain amount of fiber incorporation will enhance the strength of the specimen. Figure 20c is enlarged, and a fiber surface can be found on the fiber surface. There are a lot of uneven areas, and these areas are sliding failure patterns. Fiber produced by scraping marks is different, more like a fiber in the brick particles or quartz sand particles under the extrusion of plastic deformation, increasing the fiber surface roughness. The friction between the fiber and the matrix is indirectly increased to improve the strength of the specimen.

## 4. Conclusions

The recycling and utilization of waste brick aggregate with a particle size less than 5 mm was mainly studied. The waste brick aggregate with a diameter of less than 2 mm was mixed into the soil, with different amounts, and combined with fibers to prepare the highway sub-base material meeting the specification requirements. The 7d unconfined compressive strength and splitting tensile strength were taken as the evaluation criteria. Recycled brick powder of 0.075~0.125 mm milled from waste brick aggregate was used to replace quartz sand with different replacement rates, together with different doses of PVA fiber to prepare environmentally friendly fiber-reinforced recycled-brick-powder cement-based composite pavement material. The relationship between the mass content of recycled brick powder, the volume content of PVA fiber, and the fiber-reinforced recycled-brick-powder cementitious composites’ strength was investigated. The possibility of application of waste red brick aggregate with different particle sizes in pavement structure is verified, and the following conclusions are drawn:

(1) The red brick aggregate was mixed into the plain soil with different content levels (0%, 50%, and 100%) and different content levels of cement (3%, 4%, and 5%). After 45 comprehensive compaction tests, the optimal moisture content and maximum dry density under nine different mixing ratios were obtained.
(2) Within the scope of the experimental study, according to the obtained 7d unconfined compressive strength and splitting tensile strength, the strength of the test group when the red-brick-aggregate admixture was ≤50%, cement admixture was ≥4%, and fiber admixture was ≥0.2% followed the specification study. Therefore, combined with the careful consideration of strength and material cost, an ideal ratio for the test is 50% of the waste red brick aggregate, 4% of cement, and 0.2% of fiber.
(3) Based on the test results of the 7d unconfined compressive strength and splitting tensile strength, a regression analysis was used to fit the mathematical relationship between the PVA-fiber content and 7d compressive and splitting tensile strength. It was determined that, when the cement content is 4%; then the PVA-fiber content is within 0.3%; and the ration of plain soil to red brick aggregate = 1:0, 1:1, and 0:1, the PVA-fiber content is 0.3%, 0.3%, and 0.25%, and the 7d unconfined compressive strength is the best value. When cement content is 4%; and the PVA-fiber content is within 0.3%; and the plain soil:red brick aggregate = 1:0, 1:1, and 0:1, then the PVA-fiber content is 0.3%, 0.3%, and 0.3%, and the maximum splitting tensile strength is the best value. In the scope of the test, comprehensive consideration, when cement content is 4%, plain soil:red brick aggregate = 1:0, 1:1, and 0:1, the optimal PVA content is 0.3%, 0.3%, and 0.25%, respectively.
(4) The flexural and compressive strength of the fiber-reinforced recycled-brick-powder cement-based composite pavement material decreases with increased quartz sand replaced by recycled brick powder. However, when the replacement rate is less than 25%, the strength does not decrease significantly and is controlled within 10%, which meets the strength requirements. Therefore, the flexural and compressive strength of fiber-reinforced recycled-brick-powder cementitious composites increased first and then decreased with the content of PVA fiber. However, when the flexural and compressive strength reach the best value, the content of PVA fiber is different. For example, when the content of PVA fiber is 1% and 1.5%, the material’s compressive strength and flexural strength reach the maximum value. Therefore, the best mixing ratio of fiber-reinforced recycled-brick-powder cementitious composites as pavement material is as follows: the replacement rate of recycled brick powder is 25%, and the content of PVA fiber is 1%.
(5) The influence of fibers on the material’s mechanical properties was analyzed from the microstructure. The mechanism of fiber pull-out, fracture, and plastic damage in three different forms of damage to the material’s mechanical properties was analyzed.

## Figures and Tables

**Table 1:** Main technical indicators of cement.

Setting Time/Min		Compressive Strength/MPa		Flexural Strength/MPa		Water Requirement of Normal Consistency/%	Stability	Fineness Modulus
Initial setting	Final setting	3d	27d	3d	27d	25.4	qualified	3.2
160	210	25.8	45.2	5.6	9.4			

**Table 2:** Parameter index of fly ash.

Fineness/%	Water Requirement/%	Loss on Ignition/%	SO_3_ Content/%	Chloride Ion/%	Water Content/%	CaO Content/%	Free CaO Content/%	Stability
9.0	91	2.0	1.6	0.007	0.1	1.6	0.1	0.2

**Table 3:** Performance index of PVA fiber.

Length/mm	Density/g/cm^3^	Tensile Strength/MPa	Elongation/%	Modulus of Elasticity/GPa
6, 9	1.3	1900	8	35

**Table 4:** Technical indexes of plain soil.

Water Content/%	Particle Size > 0.075 mm/%	Liquid Limit/%	Plastic Limit/%	Plasticity Index	Type of Soil
5.5	3.7	32.89	20.96	11.93	Low liquid limit clay

**Table 5:** Mix ratio of compaction test.

Group Number	Plain Soil/g	Red Brick Aggregate/g	Cement/g	Water/g
1	2300	0	3%	10%, 12%, 14%, 16%, 18%
2	1150	1150	3%	10%, 12%, 14%, 16%, 18%
3	0	2300	3%	10%, 12%, 14%, 16%, 18%
4	2300	0	4%	10%, 12%, 14%, 16%, 18%
5	1150	1150	4%	10%, 12%, 14%, 16%, 18%
6	0	2300	4%	10%, 12%, 14%, 16%, 18%
7	2300	0	5%	10%, 12%, 14%, 16%, 18%
8	1150	1150	5%	10%, 12%, 14%, 16%, 18%
9	0	2300	5%	10%, 12%, 14%, 16%, 18%

**Table 6:** Mix ratio of unconfined-compressive- and splitting-tensile-test specimens.

Group Number		Mass of Single Specimen /g	Dry Material (Soil + Cement)/g	Inorganic Binding Material/g	Soil or Red Brick Quality/g	Water/g	Fiber Quality/g
Plain soil:red brick aggregate = 100%:0%	C3P0	199.33	176.18	5.29	170.90	23.15	0
	C4P0	204.90	178.98	7.16	171.82	25.92	0
	C5P0	208.01	180.84	9.04	171.80	27.16	0
	C3P0.1	199.33	176.18	5.29	170.90	23.15	0.17
	C4P0.1	204.90	178.98	7.16	171.82	25.92	0.17
	C5P0.1	208.01	180.84	9.04	171.80	27.16	0.17
	C3P0.2	199.33	176.18	5.29	170.90	23.15	0.34
	C4P0.2	204.90	178.98	7.16	171.82	25.92	0.34
	C5P0.2	208.01	180.84	9.04	171.80	27.16	0.34
	C3P0.3	199.33	176.18	5.29	170.90	23.15	0.51
	C4P0.3	204.90	178.98	7.16	171.82	25.92	0.52
	C5P0.3	208.01	180.84	9.04	171.80	27.16	0.52
Plain soil:red brick aggregate = 50%:50%	C3P0	188.81	165.00	4.95	160.05	23.81	0
	C4P0	190.59	165.93	6.64	159.29	24.66	0
	C5P0	191.61	166.68	8.33	158.34	24.93	0
	C3P0.1	188.81	165.00	4.95	160.05	23.81	0.16
	C4P0.1	190.59	165.93	6.64	159.29	24.66	0.16
	C5P0.1	191.61	166.68	8.33	158.34	24.93	0.16
	C3P0.2	188.81	165.00	4.95	160.05	23.81	0.32
	C4P0.2	190.59	165.93	6.64	159.29	24.66	0.32
	C5P0.2	191.61	166.68	8.33	158.34	24.93	0.32
	C3P0.3	188.81	165.00	4.95	160.05	23.81	0.48
	C4P0.3	190.59	165.93	6.64	159.29	24.66	0.48
	C5P0.3	191.61	166.68	8.33	158.34	24.93	0.48
Plain soil:red brick aggregate = 0%:100%	C3P0	154.87	139.83	4.19	135.63	15.05	0
	C4P0	157.41	140.48	5.62	134.86	16.93	0
	C5P0	162.67	143.84	7.19	136.64	18.84	0
	C3P0.1	154.87	139.83	4.19	135.63	15.05	0.14
	C4P0.1	157.41	140.48	5.62	134.86	16.93	0.13
	C5P0.1	162.67	143.84	7.19	136.64	18.84	0.14
	C3P0.2	154.87	139.83	4.19	135.63	15.05	0.27
	C4P0.2	157.41	140.48	5.62	134.86	16.93	0.27
	C5P0.2	162.67	143.84	7.19	136.64	18.84	0.27
	C3P0.3	154.87	139.83	4.19	135.63	15.05	0.41
	C4P0.3	157.41	140.48	5.62	134.86	16.93	0.40
	C5P0.3	162.67	143.84	7.19	136.64	18.84	0.41

Note: C stands for cement, and P for PVA fiber. For example, C3P0.1 represents a sample with cement content of 3% and PVA-fiber content of 0.1%.

**Table 7:** Fiber-reinforced recycled-brick-powder cementitious composites’ fitting ratio.

Group Number	Cement Quality/g	Fly Ash Quality/g	Recycled Brick Powder Quality/g	Quartz sand Quality/g	Quality of Water/g	PVA Fiber Quality/g	Water Reducer/%	Thickening Agent/%
Z0P0	439.84	527.81	0	348.36	309.66	0	0.2	0.1
Z25P0	439.84	527.81	87.09	261.27	309.66	0	0.2	0.1
Z50P0	439.84	527.81	174.18	174.18	309.66	0	0.2	0.1
Z75P0	439.84	527.81	261.27	87.09	309.66	0	0.2	0.1
Z100P0	439.84	527.81	348.36	0	309.66	0	0.2	0.1
Z0P1	439.84	527.81	0	348.36	309.66	3.33	0.2	0.1
Z25P1	439.84	527.81	87.09	261.27	309.66	3.33	0.2	0.1
Z50P1	439.84	527.81	174.18	174.18	309.66	3.33	0.2	0.1
Z75P1	439.84	527.81	261.27	87.09	309.66	3.33	0.2	0.1
Z100P1	439.84	527.81	348.36	0	309.66	3.33	0.2	0.1
Z0P1.5	439.84	527.81	0	348.36	309.66	4.99	0.2	0.1
Z25P1.5	439.84	527.81	87.09	261.27	309.66	4.99	0.2	0.1
Z50P1.5	439.84	527.81	174.18	174.18	309.66	4.99	0.2	0.1
Z75P1.5	439.84	527.81	261.27	87.09	309.66	4.99	0.2	0.1
Z100P1.5	439.84	527.81	348.36	0	309.66	4.99	0.2	0.1
Z0P2	439.84	527.81	0	348.36	309.66	6.66	0.2	0.1
Z25P2	439.84	527.81	87.09	261.27	309.66	6.66	0.2	0.1
Z50P2	439.84	527.81	174.18	174.18	309.66	6.66	0.2	0.1
Z75P2	439.84	527.81	261.27	87.09	309.66	6.66	0.2	0.1
Z100P2	439.84	527.81	348.36	0	309.66	6.66	0.2	0.1

Note: Z stands for recycled brick powder, and P for PVA fiber. For example, Z25P0 represents a recycled brick powder content of 25% and a PVA-fiber content of 0%.

**Table 8:** Compaction test results.

Group Number	Content of Plain Soil/%	Red-Brick-Aggregate Content/%	Cement Content/%	Maximum Dry Density/(g/cm^3^)	Optimum Moisture Content /%
1	100	0	3	1.89	13.14
2	50	50		1.77	14.43
3	0	100		1.50	10.76
4	100	0	4	1.92	14.48
5	50	50		1.78	14.86
6	0	100		1.51	12.05
7	100	0	5	1.94	15.02
8	50	50		1.79	14.96
9	0	100		1.54	13.10

**Table 9:** Results of the 7d-unconfined-compressive-strength and splitting-strength test.

Group Number		7d Unconfined Compressive Strength/MPa	Splitting Strength /MPa
Plain soil:red brick aggregate = 1:0	C3P0	0.62	0.06
	C4P0	1.18	0.10
	C5P0	1.39	0.14
	C3P0.1	0.73	0.09
	C4P0.1	1.52	0.15
	C5P0.1	1.62	0.19
	C3P0.2	0.79	0.10
	C4P0.2	2.04	0.17
	C5P0.2	2.23	0.21
	C3P0.3	0.96	0.11
	C4P0.3	2.18	0.21
	C5P0.3	2.38	0.23
Plain soil:red brick aggregate = 1:1	C3P0	0.85	0.08
	C4P0	1.16	0.13
	C5P0	1.35	0.17
	C3P0.1	1.29	0.13
	C4P0.1	1.79	0.20
	C5P0.1	2.51	0.26
	C3P0.2	1.36	0.15
	C4P0.2	2.16	0.23
	C5P0.2	2.72	0.32
	C3P0.3	1.47	0.18
	C4P0.3	2.76	0.29
	C5P0.3	2.93	0.38
Plain soil:red brick aggregate = 0:1	C3P0	0.32	0.04
	C4P0	0.87	0.08
	C5P0	1.13	0.12
	C3P0.1	0.50	0.05
	C4P0.1	1.20	0.12
	C5P0.1	1.31	0.13
	C3P0.2	0.66	0.07
	C4P0.2	1.28	0.13
	C5P0.2	1.47	0.15
	C3P0.3	0.84	0.08
	C4P0.3	1.33	0.15
	C5P0.3	1.55	0.16

**Table 10:** Compressive and flexural strength of fiber-reinforced recycled-brick-powder cementitious composites.

Group Number	Compressive Strength/MPa	Flexural Strength/MPa
Z0P0	37.86	14.56
Z25P0	35.63	13.12
Z50P0	31.76	12.34
Z75P0	28.32	11.74
Z100P0	25.66	10.56
Z0P1	38.12	16.47
Z25P1	36.03	15.32
Z50P1	32.31	14.47
Z75P1	28.47	13.23
Z100P1	26.34	11.96
Z0P1.5	36.47	17.56
Z25P1.5	34.32	16.14
Z50P1.5	29.61	15.37
Z75P1.5	27.54	14.43
Z100P1.5	25.21	12.54
Z0P2	34.78	16.15
Z25P2	34.97	14.23
Z50P2	28.73	13.15
Z75P2	25.43	12.65
Z100P2	25.01	11.32

## Data Availability

All data generated or analyzed during this study are included in this published article.
